# Patients Characteristics and Psychosocial Treatment in Psychodynamic and Cognitive Behavior Therapy

**DOI:** 10.3389/fpsyt.2021.664975

**Published:** 2021-05-14

**Authors:** Beate Muschalla, Michael Linden, Matthias Rose

**Affiliations:** ^1^Department of Psychotherapy and Diagnostics, Technische Universität Braunschweig, Braunschweig, Germany; ^2^Research Group Psychosomatic Rehabilitation, Department of Psychosomatic Medicine, Charité University Medicine Berlin, Berlin, Germany

**Keywords:** capacity, International Classification of Functioning Disability and Health (ICF), participation, biopsychosocial, Mini-ICF-APP, cognitive behavior counseling, psychodynamic, psychotherapy

## Abstract

**Introduction:** The most prevalent psychotherapy schools are psychodynamic (PDT) and cognitive behavior therapy (CBT). There are no scientific guidelines on which type of patient should be treated by which therapist, and how they can find the best one. Part of the answer can be derived from data on who is treated in which way.

**Objective:** Objective of this study was to compare patients in routine PDT and CBT to describe similarities and differences in regard to patient status and treatment.

**Materials and Methods:** A research psychotherapist visited 73 cognitive behavior therapists and 58 psychodynamic psychotherapists in their office and asked them to report about the last cases they had seen. There were 188 CBT and 134 PD case reports.

**Results:** There were no significant differences in socio-demographics between PDT and CBT patients. The average number of treatment session so far was significantly higher in PDT than CBT. There were longer duration of illness, more parallel medical treatments and higher rates of sick leave in CBT patients. While assessment of capacities with the Mini-ICF-APP showed no differences, more participation restrictions were found in CBT patients. Correspondingly there were more sociomedical interventions, especially in regard to work.

**Conclusions:** The differences between PDT and CBT may be explained by the fact that PDT requires analytical capabilities on the side of the patient, which may exclude patients with social problems, while CBT is coping oriented which allows the inclusion of all kinds of patients. Still, in both treatment modes, complex patients are treated with multidimensional interventions.

## Introduction

There are different psychotherapy schools, following different theoretical models and using different techniques (1, 2). They similarly aim to reduce the symptoms of illness and suffering of the patient, though following different concepts, and using different approaches and techniques (3, 4). There have been changing tendencies for predominance of certain therapy forms during the development of psychotherapy until now (5). But, most known and used are psychodynamic therapy (PDT) and cognitive behavior therapy (CBT). Their similar efficacy has well been shown in many empirical studies across a wide spectrum of disorders (6–14).

Still, there is a lack of research on which patients should best be treated in CBT and PDT. Some suggestions are that phobias might preferably need behavioral exposure therapy, and some personality disorders preferably PDT (15), but both treatments are effective (16). It was found that PDT therapists valued a neutral relationship, CBT therapists emphasized a didactic interaction (2).

But, superiority effects of the one or other therapy are often small (12). Recommendations are rather vague and do not result in guidelines which could guide patients to find the best therapy. According to theoretical frames of reference, it may be that PDT is primarily focused on inner psychodynamic conflicts, whereas CBT rather aims at symptom management and improvement of coping. Qualitative differences are also described for inter-session processes (17), or dose effects in relation to lasting outcomes (15), or effects of specific resource-oriented interventions (10).

A survey in a naturalistic setting for patients with substance use disorders in Sweden (18) found that there are more often trauma problems in PDT (22% as compared to 6% in cognitive therapy), more often anxiety problems in CBT (52 vs. 24%), that personality disorders are diagnosed in 20% of PDT and 8–29% in CBT patients, while the global assessment of functioning (GAF) is similar with a score of 54 in PDT and 55 in CBT. When comparing patients between therapies, it is also of interest to study what is done therapeutically. In 29% of PDT treatments relational improvement was the primary goal, but only in 12–16% in CBT, whereas in CBT the focus was on symptom and affect management (28–39% in CBT vs. 18% in PDT), and behavior change (29–62% in CBT vs. 9% in PDT) (18).

Apart from school-specific and symptom oriented treatment goals and techniques, there is school-overlapping a second level of targets and interventions, regarding capacity limitations and participation restrictions in life, as defined by the International Classification of Functioning Disability and Health (19). Many patients have to struggle with problems at home, at work, and in leisure time, and show high rates of work incapacity and early retirement (20–28). This must therapeutically be addressed. Treatments in this regard can be called “sociomedical treatments.” Psychotherapists should initiate and guarantee interdisciplinary cooperative treatments. They should take care of disability assessments and work related measures, or procure help in daily life problems. A comparison of such interventions in PDT and CBT can also shed some light on the question of differential patient allocation and care.

There is a lack of empirical data on the indication of different psychotherapy schools. It is therefore of interest, whether in routine care PDT and CBT therapists see different or the same patients. It could be that PDT and CBT therapists have their ideas on which type of problem they can best deal with and therefore accept different patients for treatment. It could be that patients are informed by their physicians or other therapists to go here or there. Patients may also have thoughts of their own about what is done in PDT or CBT, and what they prefer. With this given, the objective of our study was to assess the severity and chronicity of illness, capacity limitations, participation restrictions, the prognosis of treatment, and resulting sociomedical interventions in PDT and CBT.

As can be seen from the empirical evidence until now, there is a lot of research on patients characteristics and psychotherapy in PDT and CBT. What has until now been missing (and is central part of this present study) are socio-medical problems and social-oriented / socio-medical treatments in PDT and CBT. Therefore our study is of high originality and novelty. It presents first data on socio-medical aspects in psychotherapy treatments (comparing PDT and CBT).

## Materials and Methods

### Health Care System in Germany

The study was done in Berlin, Germany. Health insurance covers all citizens and fully reimburses up to 80 sessions CBT or 120 sessions PDT. Psychotherapy is provided by physicians and psychologists in private practice, who either have a state licensed school specific education in PDT or CBT, and who are obliged to provide only the treatment of their expertise. We included psychologist-psychotherapists, who were doing psychotherapy only. In Berlin there is a total of 2.051 listed, so that there is about one per 2000 inhabitants. There are furthermore physician-psychotherapists, who were not contacted, as they can do additional medical treatments, so that patient selection may not only be due to psychotherapy.

### Recruitment of Psychotherapists

Referring to official listings, psychotherapists were contacted with no special selection, but an equal number of PDT and CBT. Therapists were informed that the goal was to study which type of patients were finding their way into psychotherapy. Therapists reported on their gender, age, education, years of practice, number of patients, and predominant spectrum of disorders in their practice.

### Interviews With Psychotherapists

Therapists were interviewed in their office by researchers who were themselves psychotherapists, following a semi standardized interview. The interviewers validated the answers of the therapists by clarifying what was meant. Based on 20 parallel interviews, the interrater reliability was κ = 0.803 (95% CI, *p* < 0.0005). Therapists were asked to refer anonymously to the last patients of working age whom they had seen, with a minimum of 10 therapy sessions. In accordance with the WHO International Classification of Functioning Disability and Health [ICF, (19)] patient assessment did not only ask for symptoms (disorders of functions) and course of illness, but also for capacities and participation.

“*Disorders of functions (symptoms)”* were assessed with a Visual Analog Mood Scale [VAS, (29)] with ratings from 0 = negative to 10 = good for depression, anxiety, nervousness, somatoform symptoms, tiredness, memory problems, delusional symptoms.

“*Severity of illness”* was rated on the Clinical Global Impression scale [CGI, (30)] from normal, not at all ill (1) to among the most extremely ill patients (7).

“*Capacity limitations”* were assessed following the Mini-ICF Assessment of Activities and Participation in Psychological Disorders [Mini-ICF-APP (31, 32)]. It lists in reference to the ICF (19) capacities which are typically impaired by mental disorders. These are: (1) adherence to regulations, (2) planning and structuring of tasks, (3) flexibility, (4) competency, (5) competence to judge and decide, (6) proactivity, (7) endurance, (8) assertiveness, (9) contact with others, (10) group integration, (11) dyadic relationships, (12) self-care, and (13) mobility. Each dimension was rated on a six-point Likert-scale (0: cannot be assessed; 1: no disability; 2: mild disability; 3: moderate disability; 4: severe disability; 5: total disability). The degree of impairment per capacity was rated in reference to the workplace, or in case of unemployment, to the general labor market. A sum score gives an idea of the overall impairment.

“*Participation restrictions”* were assessed with the Index for Measuring Participation Problems (IMEP) (31, 33), covering (1) activities of daily living, (2) recreational activities, (3) social activities, (4) close relationships, (5) special stressors in life, (6) work. A judgment is made on a visual analog scale from 0 = no impairment to 10 = full impairment, i.e., no activity is possible in this domain of life.

“*Sociomedical interventions”* were coded in reference to the TOPP Checklist [(Treatment Options for Persistent Psychological Problems; (34)], listing 38 items, like cooperation with other therapists, contacts with institutions, work-related interventions, social support. Therapist are asked to indicate, whether they have done, initiated or participated in these interventions.

### Ethical Considerations

The project was approved by the ethical committee of the Charité University Medicine Berlin and by the data privacy department of the Pension Insurance Berlin-Brandenburg (EA4/027/18, 10-R-40.07.05.07.017). The therapists gave written informed consent. There was no direct involvement of patients and no interference with the ongoing treatment.

### Statistical Analysis

Data have been analyzed with SPSS, version 26. Exploratory group comparisons have been calculated with *T*-test for continuous variables and Chi^2^-Test for frequencies. Significance levels are reported for single items to guide attendance to points of special interest.

## Results

We invited 326 therapists. An interview could be done with 131 therapists. There were 74% female therapists, with an average age of 54 years (s.d. 10.97, range 32–74). They worked as psychotherapist for 16.8 years (s.d. 10.70, range 1–45). Of these, 43.5% were psychodynamic therapists, 55.7% cognitive behavior therapist, and one was trained in both. Therapists reported to treat on average 39 patients (SD ± 24; min. 5, max. 150). This includes short- and long-term treatments, as well as acute counseling sessions and sessions for relapse prevention.

Referring to the last seen patients in working age, therapists reported on 322 case vignettes (58.4% CBT, 41.6% PDT).

### Sociodemographic Characteristic of Patients

There were no differences between PDT and CBT in regard to general patient characteristics like age, gender, migration background, family status, education, employment (see Table 1).

**Table 1:** Characteristics of patients and therapy methods in cognitive behavior therapy and psychodynamic psychotherapy.

**Patient and therapy characteristics**	**PDT** ** (*n* = 134)**	**CBT** ** (*n* = 188)**	***T*-test or χ^**2**^-test** ***p***
**Sociodemographic characteristics**			
Age	41.16 (11.5)	41.97 (10.6)	0.516
Sex female	66.4%	65.4%	0.694
Migration background	15.7%	9.3%	0.263
With partner or married	42.6%	49%	0.690
Highest professional qualification			0.497
No professional qualification	22.4%	15.4%	
Master of crafts, Professional academy	2.2%	1.6%	
Professional apprenticeship	46.3%	47.9%	
University diploma	27.6%	32.4%	
Another	1.5%	2.7%	0.165
Presently employed in fulltime or part-time	60.4%	52.7%	
Work situation and interaction with the illness status (1 = work is a helpful resource; 4 = work is pathogenetic)	2.21 (1.22)	2.57 (1.13)	0.015
**Illness status**			
VAS depression	4.3 (2.2)	4.2 (2.3)	0.88
VAS anxiety	3.8 (2.2)	3.8 (2.1)	0.79
VAS nervousness	3.7 (2.1)	3.8 (2.2)	0.65
VAS somatoform symptoms	5.4 (2.9)	5.9 (2.8)	0.18
VAS lack of energy	4.9 (2.3)	4.6 (2.5)	0.38
VAS memory problems	6.9 (2.7)	6.2 (2.6)	0.02
VAS thought disorders	8.7 (2.1)	8.7 (2.1)	0.95
VAS mean	5.3 (1.7)	5.2 (1.6)	0.29
Severity of present illness (CGI) 1 = not ill at all, 7 = severely ill	4.70 (1.05)	4.83 (1.17)	0.316
Parallel medical treatment	18.7%	6.4%	0.001
Sick leave duration within the past 12 months in weeks	11.80 (17.2)	17.93 (20.5)	0.007
Duration of present illness episode in months	40.54 (59.15)	52.7 (78.8)	0.136
Number of outpatient psychotherapies before	0.74 (1.06)	0.69 (0.82)	0.120
Number of acute inpatient therapies before	0.50 (0.89)	0.99 (2.50)	0.032
Number of inpatient psychosomatic rehabilitation before	0.31 (0.65)	0.47 (0.78)	0.054
**Capacity limitations and participation restrictions**			
Mini-ICF-APP capacity limitations mean	0.97 (0.66)	1.08 (0.64)	0.144
IMEP mean	3.32 (1.57)	3.75 (1.71)	0.024
IMEP activities of daily living	2.62 (1.91)	2.86 (2.08)	0.413
IMEP recreational activities	2.86 (1.91)	3.13 (2.10)	0.296
IMEP social activities	3.07 (1.98)	3.50 (2.38)	0.123
IMEP close relations	4.19 (2.44)	3.99 (2.55)	0.202
IMEP coping with Stress	3.99 (2.12)	4.71 (2.07)	0.003
IMEP work and professional activities	3.91 (2.71)	4.97 (2.83)	0.003
**Psychotherapy process**			
How many therapy sessions have been done until now?	55.67 (58.01)	33.95 (19.76)	0.000
Good patient-therapist cooperation (rating 1–4)	3.42 (0.58)	3.53 (0.54)	0.071
No side effects of treatment	32.8%	31.9%	0.862
Prognosis of therapy (1 = complete remission 2 = persistent moderate impairment 3 = persistent severe impairment	1.90 (0.67)	2.04 (0.62)	0.055
Work ability at the end of treatment 1 = fit for work 2 = partially fit for work 3 = unfit for work	1.48 (0.63)	1.58 (0.67)	0.160
**Coordination with other therapists**			
Medical specialist	15.2%	34.8%	0.000
Inpatient psychosomatic rehabilitation	9.9%	18.0%	0.169
Inpatient psychiatric treatment	5.9%	9.6%	0.57
Self help groups	4.0%	8.1%	0.26
Psychoeducative seminars	2.8%	8.4%	0.032
Other psychotherapists	3.1%	6.8%	0.21
Occupational therapy	3.4%	4.7%	0.94
Socio-psychiatric service	2.4%	5.3%	0.31
Drug counseling	1.9%	3.1%	0.73
Post-hospital psychosomatic rehabilitation	1.6%	2.8%	0.65
Sociotherapist	1.9%	1.9%	0.55
Medical service of health insurance	0.6%	5.9%	0.002
Day care clinic	0.6%	2.5%	0.16
Drug withdrawal treatment	1.2%	1.5%	0.86
Ambulance service	0.3%	0.6%	0.77
**Work participation**			
Sick leave certification	7.8%	20.2%	0.002
Disability certification	8.7%	19.3%	0.017
Job center	5.9%	11.4%	0.199
Stepwise work reintegration	3.4%	9.6%	0.03
Early retirement pension	4.3%	7.5%	0.525
Rehabilitation counseling	3.7%	7.1%	0.35
Work rehabilitation	3.1%	2.5%	0.22
Occupational integration management	0.9%	6.2%	0.004
Works council	0.9%	5.0%	0.019
Company doctor	1.9%	7.5%	0.012
**Participation in life**			
Sport club	9.0%	18.3%	0.053
Contact with family members	2.4%	6.5%	0.11
Family counseling	3.1%	4.7%	0.87
Communal counseling center	2.2%	4.3%	0.43
Family support	2.2%	3.1%	0.97
Guardian	1.2%	3.1%	0.31
Debt counseling	1.2%	2.8%	0.42
Sheltered living	0.9%	3.1%	0.17
Individual casework	0.6%	2.5%	0.16
Psychiatric home care	0.6%	1.2%	0.68
Integration center for disabled persons	0.3%	0.9%	0.50
Home visit	0%	0.6%	0.23

### Illness Status

Both therapist groups made similar diagnoses, mostly depression (F30: PDT 26.1%, CBT 35.1%), adjustment and traumatic disorder (F43: PDT 9.3%, CBT 15.5%), somatoform disorders (F45: PDT 4.3%, CBT 11.5%), and personality disorders (PDT 6.8%, CBT 9.3%). Therapists reported no significant differences in regard to the illness status, neither in the spectrum and severity of the symptoms, as measured with the VAS and CGI. Duration of illness was similar in PDT and CBT, though with a large variance. There was a trend for more inpatient stays in CBT patients, be it in acute or rehabilitation hospitals. CBT patients are also more often in parallel medical treatment.

### Capacity Limitations

Therapists reported a similar degree of capacity limitations, be it in the global score or individual dimensions of the Mini-ICF-APP.

### Participation Restrictions

The IMEP mean score suggests that CBT therapists see more participation problems than PDT therapists. This is especially so for coping with stress and work.

### Psychotherapy Process

PDT patients are on average significantly longer in treatment than CBT patients. There is a trend that CBT therapist report a somewhat better patient therapist cooperation. There were no differences in regard to report on negative effects of treatment. CBT therapists expect a relatively poorer prognosis of therapy.

### Sociomedical Interventions

Table 1 groups sociomedical interventions, which have been effected, in the sections (a) coordination and cooperation with other therapists, (b) interventions to improve work participation, and (c) interventions to improve participation in daily life. There by and large a similar global rank order for these interventions in both treatment groups. CBT therapists report higher rates for all items. Greatest differences are found for psychoeducation courses, which are a typical behavior therapy treatment method in itself. There are foremost relevant differences in regard to work related interventions, like inability and disability certification, occupational integration management, stepwise work reintegration, and contacts with work doctors or councils.

## Discussion

A first result is that there are no major differences between CBT and PDT patients in regard to sociodemographic characteristics. Similar patients are seen in both treatments. While both therapist groups rate the severity of illness very similar, there are still indicators that in CBT more heterogeneous or complex cases may be treated. There is a longer duration of illness, a higher rate of parallel medical treatments, of sick leave, and more prior inpatient stays. CBT therapist report more participation problems as measured with the IMEP. Correspondingly, CBT therapists report more sociomedical interventions, like interdisciplinary treatment, work oriented interventions, and social support. Finally, CBT therapists expect a poorer outcome of treatment.

In summary, these findings suggest that PDT and CBT therapists attract or accept patients with different types of problems and execute different treatments. This corresponds with basic concepts of both treatment approaches. As described in the scientific literature, CBT is seen as more problem oriented which allows to treat more chronic illnesses and to apply more socio-medical interventions. CBT may highlight their client's wish to master demands in family and work. PDT is described to be more insight oriented and highlighting their patients wishes to be taken care of by their therapists. In CBT therapist are very active and the approach is rather structured while in PDT patients learn insight in their unconscious dynamics (10, 15, 17, 18, 35–37). Traditionally, PDT is also characterized by the requirement, that patients must show “analytic capability.” This may exclude to some part patients with a lack of psychological insight, with complex mental disorders, like dementia, or with severe social problems. CBT in contrast is rather focusing on coping, not only with internal psychological processes, but also with social and life problems. This allows to target a broad spectrum of mental and somatic disorders, but also problems in daily life or at work, as can be concluded from differences in sociomedical interventions. Our findings are in line with these different psychotherapy concepts.

### Limitations

When discussing the results, one has to take into account as a limitation, that we did not assess patients directly, but asked therapists about their cases and treatments. The answers therefore reflect the views of the therapists. This results in some objective data, like the information on the duration of treatment. There is other information which is a mixture of patient characteristics and the perspective of the therapist, like the expectation of treatment outcome. For our research question it seems to be appropriate and an even advantage to interview therapists on their patients and treatment, when one wants to compare PDT and CBT.

The response rate of participating therapists was 40% in this present study. Compared with similarly scoped research in the same region done with primary physicians, which achieved a response rate of 13% (27), this present rate is a quite high response rate. The participating therapists must be expected to be prototypically representative psychotherapists, i.e., especially good colleagues with high interest in additional professional learning and activities beside their routine duties. The data must be interpreted against this background: from this sample of engaged therapists, is might be concluded what could be done (but not what is routinely done) in outpatient psychotherapy.

The reported differences represent trends. When one looks at the absolute data, it can be seen, that therapists in PDT and CBT alike treat patients of all sorts with a similarly broad spectrum of therapeutic interventions.

## Conclusion

The data add information on differential indications and patient allocation and may help to further guide research on this subject. This should get more scientific attention.

## Data Availability Statement

The raw data supporting the conclusions of this article will be made available by the authors, without undue reservation.

## Ethics Statement

The project was approved by the ethical committee of the Charité University Medicine Berlin and by the data privacy department of the Pension Insurance Berlin-Brandenburg (EA4/027/18, 10-R-40.07.05.07.017). The therapists gave written informed consent. There was no direct involvement of patients and no interference with the ongoing treatment. The patients/participants provided their written informed consent to participate in this study.

## Author Contributions

ML designed the study and research question and supervised the research process. BM analyzed the data and wrote the manuscript. MR and ML reviewed and contributed to the manuscript. All authors contributed to the article and approved the submitted version.

## Conflict of Interest

The authors declare that the research was conducted in the absence of any commercial or financial relationships that could be construed as a potential conflict of interest.
